# Perioperative Factors Associated With Allogeneic Blood Transfusion in Elective THA

**DOI:** 10.1111/os.70270

**Published:** 2026-02-10

**Authors:** Nikolai Ramadanov, Dakota Fuchs, Maximilian Heinz, Robert Prill, Roland Becker

**Affiliations:** ^1^ Center of Orthopaedics and Traumatology Brandenburg Medical School, University Hospital Brandenburg/Havel Brandenburg Germany; ^2^ Faculty of Health Science Brandenburg Brandenburg Medical School Theodor Fontane Brandenburg Germany

**Keywords:** blood transfusion, hematocrit, hidden blood loss, predictors, total hip arthroplasty

## Abstract

**Background:**

Identifying predictors of perioperative blood transfusion is essential for optimizing patient safety and perioperative blood management in total hip arthroplasty (THA). This Study aimed to identify perioperative factors independently associated with transfusion.

**Methods:**

This retrospective study included all elective primary THA procedures performed between 2016 and 2023 at a certified Endoprosthetic Center. Demographic, clinical, laboratory, and operative variables were extracted. Total and hidden blood loss were calculated using the Nadler and Gross/Sehat formulas. A multivariable logistic regression model was fitted to identify independent factors associated with transfusion. Receiver operating characteristic (ROC) analyzes were performed for cup inclination and the 48‐h hematocrit. Cutoff thresholds were derived using Youden's index, and combined decision rules (AND/OR) were evaluated.

**Results:**

Among 39 predictors, three variables were independently associated with transfusion: cup inclination (OR = 0.89; *p* = 0.0003), 48‐h hematocrit (OR ≈5.17 × 10^−48^; *p* = 0.023), reflecting quasi‐separation due to a near‐deterministic threshold effect rather than a literal effect size, and reoperation (OR = 13.19; *p* = 0.049). The model demonstrated excellent discrimination (AUC = 0.931). Inclination alone showed negligible predictive value (AUC = 0.393). The 48‐h hematocrit was a strong single predictor (AUC = 0.817) with a clinically meaningful threshold (< 0.28 L/L). Combined rules showed moderate performance; the OR rule was ineffective.

**Conclusion:**

Early postoperative hematocrit is a robust and clinically actionable marker associated with transfusion risk in primary THA. Cup inclination reflects the surgical technique rather than direct transfusion risk, and reoperation likely indicates underlying perioperative complexity. Transfusion strategies should prioritize hematocrit‐based evaluation rather than inclination‐based thresholds.

**Level of Evidence:**

III—retrospective single‐center observational cohort study.

## Graphical abstract

Early postoperative hematocrit is the strongest and most clinically useful predictor of transfusion risk after primary THA, while cup inclination has minimal discriminative value. A hematocrit threshold of < 0.28 L/L reliably identifies high‐risk patients and should guide transfusion‐related decision‐making.

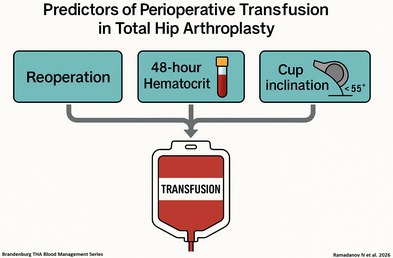

**Abbreviations**
ASA: American Society of Anesthesiologists
AUC: Area Under the Curve
BMI: Body Mass Index
CI: Confidence Interval
CS: Cannulated Screw
DHS: Dynamix Hip Screw
DVT: Deep Vein Thrombosis
EPZ: Endoprosthetic Center
FPR: Wrong Positive Rate
GT: Great Trochanter
HBL: Hidden Blood Loss
Hb: hemoglobin
Hct: hematocrit
HHS: Harris Hips Score
MCH: mean corpuscular hemoglobin
MCHC: mean corpuscular hemoglobin concentration
MCV: mean corpuscular volume
NPV: Negative Predictive Value
OR: Odds Ratio
PBM: Patient Blood Management
PE: Pulmonary Embolism
PPV: Positive Predictive Value
PRBC: Packed Red Blood Cells
RBC: red blood cells
RCT: Randomized Controlled Trial
ROC: Receiver Operating Characteristic
SE: Standard Error
STROBE: Strengthening the Reporting of Observational Epidemiology
THA: total hip arthroplasty
TRALI: transfusion‐related acute lung injury
TXA: tranxeamic acid

## 1 Introduction

Total hip arthroplasty (THA) is one of the most successful orthopedic interventions, reliably improving quality of life through pain relief and restoration of mobility. Nonetheless, substantial perioperative blood loss, both overt and hidden, remains a frequent and clinically important challenge. The resulting decline in hemoglobin often necessitates allogeneic blood transfusion, which, while sometimes life‐saving, is associated with significant risks including immunologic reactions, infection, transfusion‐related acute lung injury (TRALI), prolonged hospitalization, and even increased mortality [1]. Identifying patients at greatest risk for transfusion is therefore essential to improve perioperative planning and support blood conservation strategies.

A wide range of predictors of transfusion risk has been described. Systematic reviews and large database analyzes consistently highlight patient and procedure related factors such as preoperative anemia, female sex, advanced age, low body mass index (BMI), high American Society of Anesthesiologists (ASA) classification, surgical drainage, and conversion from prior procedures as important contributors [2, 3, 4, 5]. Medication use also plays a role: continuation of antiplatelet therapy markedly increases transfusion likelihood, whereas administration of tranexamic acid (TXA) has been shown to significantly reduce perioperative blood loss and transfusion requirements [6, 7].

In this context, Patient Blood Management (PBM) programs have been developed as multimodal approaches to reduce dependence on transfusion. Preoperative hemoglobin optimization in particular has demonstrated benefit, leading not only to decreased transfusion demand but also to shorter hospital stays and more favorable discharge outcomes [8, 9]. Despite these advances, preoperative anemia remains highly prevalent, affecting approximately one in five patients undergoing joint replacement, with even higher rates reported in revision surgery [10].

Yet, considerable heterogeneity persists in reported transfusion rates and thresholds across studies and institutions, limiting generalizability. More importantly, few large‐scale investigations have integrated both patient‐specific and surgical variables to determine which factors most strongly predict transfusion in modern elective THA cohorts.

The aims of this retrospective study were threefold: (1) to identify perioperative factors independently associated with allogeneic blood transfusion in elective primary THA within a contemporary PBM‐guided setting; (2) to compare the relative predictive and discriminatory performance of patient‐specific laboratory markers versus procedural and technical variables; and (3) to evaluate the clinical utility and limitations of cutoff‐based decision rules for early postoperative transfusion risk stratification.

## 2 Methods

### 2.1 Study Context

This retrospective analysis was conducted in accordance with STROBE guidelines [11] and forms part of the *Brandenburg THA Blood Management Series*. All elective primary THAs performed at the Endoprosthetic Center (EPZ) of the University Hospital Brandenburg/Havel between 1 January 2016 and 31 December 2023 were identified through the institutional registry (> 1400 cases). Ethical approval for all retrospective evaluations was granted by the Ethics Committee of the University of Brandenburg (No. 292032025‐BO‐E‐RETRO).

### 2.2 Data Extraction

Two investigators (NR, DF) independently extracted data from the electronic hospital information system. Operative reports, anesthesia records, discharge summaries, and internal quality assurance forms were reviewed. Extracted variables included demographics (age, sex, height, weight), BMI (calculated), ASA class, surgical indication, surgeon experience, operative time, cement use, intra‐ and postoperative complications, reoperations, transfusion events (yes/no, number of RBC units), and laboratory values (RBC, Hb, Hct, MCV, MCH, MCHC). Postoperative labs were captured at predefined intervals (≤ 48 h, postoperative days 3–6, ≥ 7 days) and immediately before/after transfusion. During the entire study period (2016–2023), transfusion decisions followed institutional Patient Blood Management–oriented standards, primarily based on hemoglobin thresholds in combination with clinical symptoms (e.g., hemodynamic instability, signs of hypoxia), rather than isolated laboratory values. These criteria were applied consistently across treating physicians as part of routine clinical practice. Cup inclination was measured on standardized postoperative anteroposterior pelvic radiographs according to institutional routine. All procedures were performed within a certified Endoprosthetic Center following standardized perioperative workflows by experienced surgeons. Surgeon experience was operationalized as a categorical binary variable based on institutional certified Endoprosthetic Center classification, distinguishing procedures performed by the designated primary consultant surgeon (“Hauptoperateur”) from those performed by non‐primary surgeons. Surgeon experience was not modeled as a continuous variable, nor quantified by years of practice or cumulative case volume. Disagreements in data extraction were resolved by consensus. Missing predictor values were handled by complete‐case analysis. Cases with missing data required for the calculation of hidden blood loss (HBL) were excluded, resulting in a complete‐case analysis for all variables included in the regression model.

### 2.3 Blood Loss Calculations

Individual blood volume was calculated using the Nadler formula [12]. Total blood loss was estimated using the Gross/Sehat dilution method. Measured blood loss was taken from the intraoperative suction volume. HBL was defined as:

$$\text{Total blood loss}–\left(\text{suction loss}+\text{transfused volume}\right).$$

One packed red blood cell (PRBC) unit was assumed to equal 260 mL unless documented otherwise.

### 2.4 Logistic Regression Analysis

A multivariable logistic regression model was fitted to identify independent factors associated with allogeneic red blood cell transfusion. All available demographic, laboratory, and procedural variables were entered simultaneously without prior feature reduction. The dependent variable was transfusion (yes/no). Continuous variables were used as linear terms; categorical variables were dummy‐coded. Odds ratios (OR) with 95% confidence intervals (CI) were computed. Multicollinearity diagnostics were not formally performed. Model discrimination was evaluated using the area under the receiver operating characteristic curve (AUC). Formal multicollinearity diagnostics (e.g., variance inflation factors) and alternative regression strategies were not applied, as the model was intended for exploratory risk discrimination rather than coefficient optimization or causal inference. Given the inclusion of multiple biologically related hematologic variables, some degree of multicollinearity was expected and individual coefficient estimates were therefore interpreted with caution.

### 2.5 ROC Analysis and Cutoff Selection

Univariate receiver operating characteristic (ROC) analyzes were conducted for two single predictors: cup inclination (degrees) and 48‐h hematocrit (l/l). Optimal cutoffs were identified using Youden's index and then clinically rounded. Sensitivity, specificity, positive predictive value (PPV), and negative predictive value (NPV) were computed for each threshold.

Two combined decision rules were evaluated using the rounded thresholds:

- **AND rule**: inclination < tIncl **and** Hct < tHct
- **OR rule**: inclination < tIncl **or** Hct < tHct

## 3 Results

### 3.1 Cohort and Descriptive Characteristics

**FIGURE 1:**
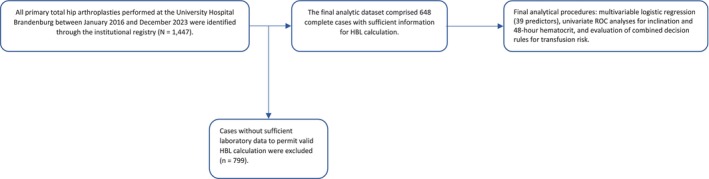
Flow diagram illustrating case selection for the final analysis. From all elective primary total hip arthroplasties performed during the study period, procedures lacking sufficient data to calculate hidden blood loss were excluded. This resulted in a final analytic sample of 648 complete cases. The flow structure aligns with the Statement Guidelines for Reporting Observational Studies (STROBE) for transparent reporting of inclusion and exclusion steps in observational cohort studies. HBL, hidden blood loss; ROC, receiver operating characteristic.

A total of 648 primary THA were included in the final analysis after exclusion of cases lacking sufficient data to calculate HBL, resulting in a complete‐case analytic cohort (Figure 1). Overall, 104 patients (16.0%) received at least one allogeneic red blood cell transfusion during the index hospital stay, while 544 patients (84.0%) were not transfused. The cohort had a mean age of 70.8 ± 10.3 years (median 71.5), with women accounting for 57.6% of all patients. Uncemented implants were used in 84.6% of procedures, and standard straight stems in 66.5%. Primary osteoarthritis was the dominant indication, accounting for 91.4% of cases. The mean duration of surgery was 72.0 ± 22.9 min, and mean intraoperative measured blood loss was 529.8 ± 311.1 mL. Calculated total blood loss averaged 1372 ± 504 mL (median 1335), while mean HBL amounted to 854.9 ± 443 mL (median 824). Postoperative complications were infrequent, with a reoperation rate of 2.0%. A detailed summary of baseline variables is presented in Table 1.

**TABLE 1:** Baseline characteristics of the analyzed patient cohort (*N* = 648) undergoing elective primary THA.

Parameter	Values
Preoperative data	
Patient age (years)	70.82 ± 10.32
	Median: 71.5 (28; 93)
Sex	Male: 275 (42.4%)
	Female: 373 (57.6%)
Operator	Senior: 619 (95.5%)
	Trainee: 29 (4.5%)
Cement	Uncemented: 548 (84.6%)
	Cemented: 100 (15.4%)
Stem	Standard straight stem: 431 (66.5%)
	Short stem: 217 (33.5%)
Indication	OA: 592 (91.4%)
	Post‐traumatic OA: 15 (2.3%)
	ANFH: 23 (3.5%)
	DDH: 17 (2.6%)
	Cancer: 1 (0.2%)
Weight	83.24 ± 18.43
	Median: 80 (41; 160)
Height	168 ± 9.3
	Median: 168 (139; 195)
BMI	29.37 ± 5.36
	Median: 28.72 (16.42365006; 49.39307851)
ASA	1: 39 (6%)
	2: 335 (51.8%)
	3: 270 (41.7%)
	4: 3 (0.5%)
Perioperative data	
Operative time	72.04 ± 22.94
	Median: 68 (36; 277)
Intraoperative blood loss	529.8 ± 311.1
	Median: 500 (50; 3000)
Total blood loss	1372 ± 504
	Median: 1335 (0; 3079)
HBL	854.9 ± 443
	Median: 824 (0; 2579)
Inclination	41.08 ± 5.15
	Median: 41 (22; 57)
Complications	
DVT/PE	No: 646 (99.7%)
	Yes: 2 (0.3%)
Infection	No: 643 (99.2%)
	Yes: 5 (0.8%)
Dislocation	No: 646 (99.7%)
	Yes: 2 (0.3%)
GT avulsion	No: 644 (99.4%)
	Yes: 4 (0.6%)
Periprosthetic fracture	No: 640 (98.8%)
	Yes: 8 (1.2%)
Nerve injury	No: 648 (100%)
	Yes: 0 (0%)
Death and reoperation	
Death	No: 646 (99.7%)
	Yes: 2 (0.3%)
Reoperation	No: 635 (98%)
	Yes: 13 (2%)

*Note*: Continuous variables are presented as mean ± standard deviation or median (range), while categorical variables are shown as absolute numbers and percentages. Total perioperative blood loss averaged 1372 ± 504 mL, and calculated HBL averaged 854.9 ± 443 mL (median 824 mL). Uncemented implant fixation accounted for 84.6% of procedures, and the overall reoperation rate was low at 2.0%.

Abbreviations: ANFH, avascular necrosis of the femoral head; ASA, American Society of Anesthesiologists; BMI, body mass index; DDH, developmental dysplasia of the hip; DVT, deep vein thrombosis; GT, greater trochanter; HBL, hidden blood loss; OA, osteoarthritis; PE, pulmonary embolism; THA, total hip arthroplasty.

### 3.2 Results of Multivariable Logistic Regression

**FIGURE 2:**
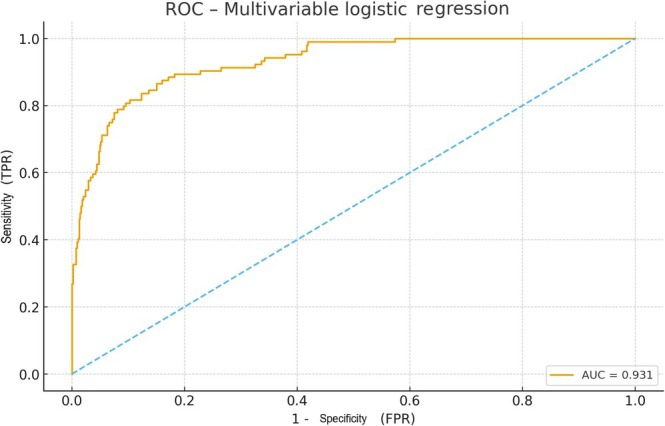
ROC curve of the multivariable logistic regression model predicting perioperative transfusion. The model demonstrated excellent discriminatory performance with an AUC of 0.931, indicating strong separation between transfused and non‐transfused patients. The diagonal line represents the line of no discrimination. AUC, area under the curve; FPR, false positive rate; ROC, receiver operating characteristic; TPR, true positive rate.

The multivariable logistic regression model included 39 predictors (28 continuous and 11 categorical variables) and was based on 104 transfusion events, corresponding to an approximate events‐per‐variable ratio of 2.7. Accordingly, the model was intended for exploratory risk discrimination rather than precise coefficient estimation or causal inference (Table 2). Cup inclination showed a protective effect: each additional degree of inclination was associated with an 11% reduction in the odds of receiving a transfusion (OR = 0.89, 95% CI 0.84–0.95; *p* = 0.0003). However, when considered as a standalone predictor, cup inclination demonstrated poor discriminatory performance (AUC = 0.393), indicating limited clinical utility despite statistical significance in the multivariable model. In contrast, the 48‐h hematocrit demonstrated a very strong inverse relationship with transfusion risk, reflected by an extremely small odds ratio (OR ≈5.17 × 10^−48^, 95% CI 6.38 × 10^−89^–4.20 × 10^−7^; *p* = 0.023), indicating that lower hematocrit values at 48 h were strongly predictive of transfusion. Reoperation during the hospital stay was also significantly associated with transfusion, increasing the odds more than 13‐fold (OR = 13.19, 95% CI 1.01–171.40; *p* = 0.049). Overall, the model demonstrated excellent discriminatory performance (AUC = 0.931), which should be interpreted as exploratory (Figure 2).

**TABLE 2:** Full multivariable logistic regression model for perioperative transfusion.

Predictor	Logistic regression coefficient β	SE	*z*‐value	*p*	OR	Lower 95% CI	Upper 95% CI
Inclination (°)	−0.115052777	0.032090486	−3.58526132	0.000336741	0.891319101	0.836985131	0.949180232
48 h postoperative Hct (l/l)	−108.8803753	48.06025434	−2.265497276	0.023482181	5.17433E‐48	6.38104E‐89	4.19582E‐07
Reoperation (Yes/No)	2.579247334	1.308561414	1.971055624	0.048717514	13.18720885	1.014619932	171.3966697
ASA_Patient with severe systemic disease that is a constant threat to life	3.651003762	1.887624795	1.934178748	0.053091154	38.51330483	0.952492684	1557.255686
ASA_Patient with severe systemic disease	1.884780373	0.999549204	1.885630407	0.059344777	6.584908058	0.928393071	46.70544782
Intraoperative blood loss (mL)	0.018489964	0.010286447	1.797507364	0.07225511	1.018661962	0.998330294	1.039407698
Total blood loss (mL)	−0.018190248	0.010774986	−1.68819228	0.091374325	0.981974196	0.961453736	1.002932628
HBL (mL)	0.016319789	0.010376084	1.572827381	0.115758794	1.016453685	0.995991095	1.037336677
Erythrozyten 48 h postoperativ in 10^12^/L	4.954798051	3.391322665	1.461022303	0.144009321	141.8539562	0.184137553	109279.9628
Indication—posttraumatic	2.260503959	1.547468824	1.460775121	0.144077166	9.587919866	0.461866586	199.0362804
Patient age (years)	0.043423363	0.031517356	1.377760312	0.168277303	1.044379953	0.981817807	1.110928604
Operative time (min.)	0.012150427	0.008952752	1.357172261	0.174726482	1.012224544	0.994617891	1.030142868
Indication—osteoarthritis	1.823401795	1.349364532	1.351304078	0.176598046	6.1928896	0.439857667	87.19157241
Indication—dysplasia	2.001032904	1.62597923	1.230663262	0.218448834	7.396692226	0.305493179	179.0909245
Cement usage_(Yes/No)	−0.504130225	0.424243473	−1.188304022	0.234713654	0.604030718	0.262991872	1.38731705
WHO BMI_obesity class III	2.826725392	2.38857202	1.183437371	0.236635854	16.89006184	0.156485842	1823.003191
ASA_Patient with mild systemic disease	1.121016224	0.970183684	1.155468025	0.247898819	3.067970366	0.458172809	20.54343249
Infection (Yes/No)	−2.424115955	2.258068957	−1.073534955	0.283031164	0.088556372	0.001059615	7.401020341
DVT/PE (Yes/No)	2.06707349	2.008875232	1.028970569	0.303493505	7.901664945	0.154085195	405.2064111
Periprosthetic fracture (Yes/No)	1.109825567	1.175583027	0.944063959	0.345136965	3.033829148	0.302923546	30.38429797
Age groups_Young	0.541549338	0.648746789	0.834762263	0.403851577	1.718667597	0.481922964	6.129233362
Height	0.218319986	0.283654394	0.769668973	0.44149628	1.243985062	0.713457499	2.169013342
Preoperative Erythrozyten in 10^12^/L	−2.021163128	2.701073134	−0.748281526	0.454290365	0.13250126	0.000665369	26.3862472
WHO BMI_obesity class II	1.141074892	1.627689601	0.701039615	0.483278293	3.130131109	0.128845907	76.04215731
Stem standard (Yes/No)	0.286457382	0.51211591	0.559360444	0.575915752	1.331701413	0.488082243	3.633462757
Nadler's total blood volume (mL)	−4.930916513	8.825035819	−0.558741813	0.576337939	0.007219883	2.2215E‐10	234646.6055
WHO BMI_obesity class I	0.577521313	1.041456495	0.554532346	0.579214575	1.781616883	0.231379535	13.71840738
Weight	0.158938606	0.315380519	0.503958225	0.614290733	1.172265975	0.631791495	2.175096571
Preoperative Hemoglobin in g/L	0.082502322	0.166986989	0.494064374	0.621260717	1.086001196	0.782871928	1.506502603
Const	−32.1423	71.27517341	−0.450960671	0.652017895	1.09844E‐14	2.35093E‐75	5.13232E+46
Sex_male (Yes/No)	1.28896981	3.618544792	0.356212202	0.721681654	3.629046022	0.003017738	4364.187356
Preoperative MCV in fl	0.147745003	0.429025187	0.344373727	0.730565245	1.159217261	0.500009134	2.687520222
48 h postoperative MCH in pg	−0.550938217	1.727852062	−0.318857285	0.749834736	0.57640876	0.019497558	17.04044429
BMI (kg/m^2^)	−0.104337583	0.332454119	−0.313840548	0.753642146	0.90092111	0.469571039	1.7285113
48 h postoperative MCHC in g/l	0.04295772	0.157188816	0.273287384	0.784632313	1.043893758	0.767108753	1.420547184
WHO BMI_overweight	−0.158290859	0.602184853	−0.26286091	0.792657786	0.853601469	0.262225231	2.778662692
48 h postoperative MCV in fl	0.116382883	0.580688513	0.200422223	0.841150381	1.12342593	0.359966003	3.506125053
GT avulsion_Yes	−1.630280424	8.379076698	−0.19456564	0.845733004	0.195874638	1.44443E‐08	2656202.758
Preoperative MCHC in g/l	−0.018255693	0.119630734	−0.152600357	0.878713444	0.981909933	0.776679403	1.24137078
Hemoglobin 48 h postoperativ in g/L	−0.00740575	0.049849202	−0.148563057	0.881898426	0.992621605	0.900226942	1.094499181
WHO BMI_underweight	−0.313464751	2.245946753	−0.139569093	0.889000462	0.730910142	0.008955929	59.65094528
Preoperative MCH in pg	0.120230624	1.362845202	0.088220308	0.929701577	1.127756909	0.078011667	16.30314666
Preoperative Haematokrit l/l	0.624365761	52.94448373	0.011792839	0.990590894	1.867061419	1.60226E‐45	2.17563E+45
Operator_trainee (Yes/No)	−32.54320049	14206.3677	−0.002290747	0.99817225	7.35644E‐15	0	inf
Dislocation (Yes/No)	−26.49522696	73548.91203	−0.00036024	0.99971257	3.11365E‐12	0	inf
Death (Yes/No)	21.27430768	88859.49368	0.000239415	0.999808974	1735059733	0	inf
Indication – cancer (Yes/No)	−17.47234785	131010.6979	−0.000133366	0.999893589	2.5814E‐08	0	inf

*Note*: This table presents all 39 predictors included in the final model, along with logistic regression coefficients (β), standard errors (SE), Wald z‐statistics, *p*‐values, OR, and 95% CI. Significant predictors (cup inclination, 48‐h postoperative hematocrit, and reoperation during the hospital stay) demonstrated independent associations with transfusion risk, while remaining variables did not reach statistical significance but are shown here for transparency and completeness.

Abbreviations: ASA, American Society of Anesthesiologists; BMI, body mass index; CI, confidence interval; DVT, deep vein thrombosis; Hct, hematocrit; MCH, mean corpuscular hemoglobin; MCHC, mean corpuscular hemoglobin concentration; MCV, mean corpuscular volume; OR, odds ratio; PE, pulmonary embolism; SE, standard error.

### 3.3 Results of Univariate ROC Analyses

When considered as a single predictor, cup inclination yielded an AUC of 0.393, indicating poor discriminatory capability. The Youden‐derived cutoff of < 55° produced very high sensitivity (0.981) but almost negligible specificity (0.0097), resulting in a Youden index of 0.0097 and confirming that inclination alone does not meaningfully distinguish between patients who did and did not receive transfusions. For clinical interpretability, the cutoff was rounded to 55°, which corresponds to commonly accepted target ranges for acetabular cup inclination in THA and reflects routine institutional practice rather than a data‐driven optimization alone.

In contrast, the 48‐h hematocrit showed strong predictive ability with an AUC of 0.817. A threshold of < 0.28 L/L provided a reasonable balance of sensitivity (0.710) and specificity (0.866), yielding a Youden index of 0.575. Positive predictive value at this threshold was 0.535, while the negative predictive value was high at 0.928, indicating that a hematocrit ≥ 0.28 L/L strongly predicted absence of transfusion.

### 3.4 Results of Combined Decision Rules

Using clinically rounded thresholds, the AND rule—defined as cup inclination < 55° and 48‐h hematocrit < 0.28 L/L—demonstrated moderate sensitivity (0.573) and high specificity (0.887), yielding a Youden index of 0.460. The OR rule, which classified patients as high‐risk when either predictor was below threshold, resulted in high sensitivity but extremely poor specificity, producing a Youden index near zero. This indicates that combining the rules with an OR logic does not improve clinical discrimination, whereas the AND rule retains practical diagnostic value (Table 3).

**TABLE 3:** Summary of univariate ROC characteristics for cup inclination and 48‐h hematocrit, including Youden‐derived thresholds, sensitivity, specificity, positive and negative predictive values, and model performance of combined AND‐ and OR‐based decision rules.

Predictor/rule	Optimal threshold	AUC	Sensitivity	Specificity	PPV	NPV	Youden J
Inclination (°)	< 55°	0.393	0.981	0.0097	0.17	0.71	0.010
48 h hematocrit (l/l)	< 0.28 l/l	0.817	0.710	0.866	0.54	0.93	0.575
Combined rule (OR)	Incl < 55° or Hct < 0.28	—	0.981	0.0097	—	—	≈0
Combined rule (AND)	Incl < 55° and Hct < 0.28	—	0.573	0.887	—	—	0.460

*Note*: Inclination < 55° showed high sensitivity but nearly zero specificity, confirming poor discriminatory value as a standalone predictor. In contrast, hematocrit < 0.28 L/L achieved balanced sensitivity and specificity and a strong Youden index. The combined AND‐rule (inclination < 55° AND hematocrit < 0.28 L/L) yielded moderate sensitivity and high specificity, providing improved clinical discrimination compared with inclination alone.

Abbreviations: AND, logical conjunction; AUC, area under the curve; OR, logical disjunction; PPV, positive predictive value; NPV, negative predictive value.

## 4 Discussion

### 4.1 Summary of Key Findings

In this retrospective evaluation of 8 years of elective primary THA at a certified EPZ, the 48‐h hematocrit emerged as the strongest and most clinically meaningful predictor of perioperative allogeneic transfusion. Cup inclination exhibited statistical significance only within the multivariable model but demonstrated negligible discriminatory ability when examined as an isolated predictor. Reoperation showed a strong association with transfusion, which likely reflects common underlying procedural complexity rather than a direct preoperative risk variable.

### 4.2 Interpretation of 48‐h Hematocrit as the Primary Transfusion Predictor

The 48‐h hematocrit emerged as the most powerful and clinically meaningful predictor of perioperative transfusion, outperforming both inclination and all other demographic and procedural variables. This strong predictive value is physiologically plausible: hematocrit reflects cumulative perioperative blood loss (visible and hidden), hemodilution, and individual patient reserve. The AUC of 0.817 and the optimal cutoff of < 0.28 L/L demonstrate that early postoperative hematocrit accurately captures the net result of intraoperative bleeding, fluid shifts, and ongoing postoperative loss. The high negative predictive value (0.928) further highlights its practical utility, as a hematocrit ≥ 0.28 L/L reliably excludes transfusion need in most cases. Unlike inclination, which operates as a surrogate of technical execution, or reoperation, which reflects postoperative course, hematocrit directly reflects the patient's physiologic state. Its superior discriminative performance confirms that transfusion decisions should be guided primarily by early postoperative hematocrit values rather than radiographic or purely procedural metrics.

Current international transfusion and Patient Blood Management (PBM) guidelines [13] primarily base transfusion decisions on hemoglobin thresholds in combination with clinical symptoms rather than on hematocrit values alone. In this context, the 48‐h hematocrit identified in the present study should not be interpreted as an alternative transfusion trigger. Instead, it represents an early postoperative marker that integrates cumulative perioperative blood loss, hemodilution, and individual patient reserve. As such, it complements guideline‐based decision‐making by aiding early risk stratification, while definitive transfusion decisions should remain aligned with established guideline recommendations.

### 4.3 Interpretation of Inclination and Surgical Quality Markers

Despite statistical significance in the multivariable model, cup inclination demonstrated negligible standalone discriminative ability (AUC 0.393) and should therefore not be interpreted as a clinically useful predictor when evaluated in isolation. The conflicting statistical behavior of cup inclination, significant in multivariable analysis yet uninformative as a stand‐alone threshold, suggests that inclination functions primarily as a surrogate indicator of surgical technique rather than a biomechanically independent determinant of blood loss. A flatter, controlled inclination, as long as it is not pathologically shallow, typically corresponds with gentler exposure, balanced soft tissue tension, and more refined acetabular preparation. These technical nuances can reduce intraoperative bleeding. Thus, inclination contributes only in the context of multivariable relationships, reflecting operative quality rather than representing a direct causal predictor.

### 4.4 Interpretation of Reoperation as Marker of Operative Difficulty

Reoperation represents a downstream postoperative outcome and should be interpreted as a marker of underlying perioperative complexity rather than a preoperative predictor. Reoperation in this dataset refers to subsequent revision surgery or hematoma evacuation, often occurring days to months after the primary procedure. Its strong association with transfusion likely arises from shared underlying intraoperative difficulty. Cases that later required revision frequently represent technically demanding or complication‐prone surgeries characterized by increased preoperative deformity, greater implant manipulation, abnormal anatomy, or surgeon‐related complexity. Consequently, transfusion and future revision surgery may represent parallel outcomes of the same initial surgical stressors rather than an independent predictive pathway.

### 4.5 Value of Cutoff‐Based Decision Rules

Cutoff‐based decision rules, although attractive for clinical simplicity, demonstrated clear limitations in this study. The poor performance of the OR‐based decision rule is explained by the opposing discriminative properties of the two variables. Cup inclination showed extremely high sensitivity but almost no specificity, whereas 48‐h hematocrit demonstrated balanced sensitivity and specificity. When combined using an OR logic, the low‐specificity component dominates, resulting in a high false‐positive rate and a Youden index close to zero. In contrast, the AND rule mitigates this effect by requiring concordance of both criteria, thereby improving specificity and overall clinical usefulness. The inclination threshold (< 55°) exhibited excellent sensitivity but essentially zero specificity, confirming that cup position alone cannot be used to guide perioperative blood management decisions. In contrast, the 48‐h hematocrit threshold (< 0.28 L/L) preserved both sensitivity and specificity, producing a Youden index of 0.575 and demonstrating strong negative predictive ability. Combined decision rules revealed that an AND logic could modestly improve clinical precision, whereas an OR logic offered no meaningful benefit. These observations highlight that only hematocrit retains clinical utility as a threshold‐based predictor, whereas inclination should not be reduced to a binary risk rule.

### 4.6 Relationship to Relevant Evidence

Sehat et al. [14] showed that “hidden” blood loss constitutes a substantial proportion of total perioperative blood loss after arthroplasty and should be considered when estimating true blood loss. Carson et al. [15] summarized randomized trial evidence supporting restrictive red blood cell transfusion thresholds (including around 8 g/dL in orthopedic surgery) and emphasized integrating hemoglobin with clinical context. Rosencher et al. [16] reported wide variability in European blood management practices and demonstrated that computed blood loss markedly exceeded estimated intraoperative loss, with baseline hemoglobin strongly influencing allogeneic transfusion probability. Goodnough and Shander [17] outlined patient blood management as a multidisciplinary, evidence‐based strategy to optimize anemia management and minimize transfusion exposure and related risks. Patel et al. [18] found that topical TXA achieved similar reductions in perioperative hemoglobin decline as intravenous TXA in a randomized arthroplasty setting, supporting pharmacologic blood‐sparing approaches.

For primary THA approaches, a 20‐RCT analysis (*n* = 1501) found SuperPATH to bleed ~82 mL less than posterior approach (MD −81.75 mL), while direct anterior approach bled ~92 mL more than posterior approach (MD +91.87 mL) and ~174 mL more than SuperPATH in indirect comparison (MD +173.62 mL); reported study‐level means spanned 89–1108 mL for SuperPATH, 166–391 mL for direct anterior approach, and 123.8–844.6 mL for posterior approach [19]. Beyond approach and procedure, meta‐regression across RCTs of SuperPATH vs. conventional hemiarthroplasty identified intraoperative blood loss (alongside age, mobilization timing, and incision length) as a significant predictor of early Harris Hip Score (HHS), reinforcing the causal plausibility that greater bleeding degrades early function [20]. Taken together, these quantitative gradients (tens to hundreds of milliliters across techniques) are consistent with our finding that early postoperative hematocrit is an effective proxy for cumulative perioperative bleeding, whereas a simple inclination cutoff adds little discrimination unless interpreted as a surrogate of operative technique quality.

### 4.7 Clinical Implications

**FIGURE 3:**
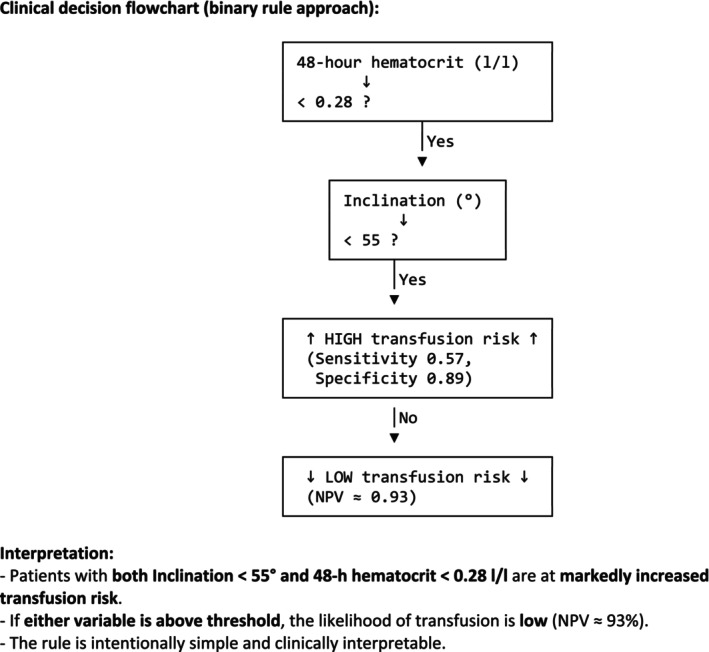
Combined cutoff‐based decision rule for transfusion risk after primary THA. This flowchart illustrates the clinically rounded thresholds derived from ROC analysis. Patients meeting both criteria—cup inclination < 55° AND 48‐h hematocrit < 0.28 L/L—show a markedly elevated transfusion risk. In contrast, patients above either threshold demonstrate a low transfusion probability, with a negative predictive value of 0.93. The visualized rule highlights practical bedside applicability and supports rapid stratification in EPZ‐standard perioperative workflows. EPZ, endoprosthetic center; THA, total hip arthroplasty.

The findings have immediate relevance for perioperative blood management in THA performed within EPZ‐certified environments: (1) routine early postoperative hematocrit measurement (≤ 48 h) should be emphasized as the primary indicator for transfusion risk assessment; (2) cup inclination should not be used as an independent trigger for transfusion decisions; its predictive value only emerges when interpreted in relation to overall surgical technique quality; (3) enhanced intraoperative vigilance is warranted in technically challenging cases, as both transfusion and future reoperation appear to originate from underlying surgical difficulty rather than isolated risk variables; (4) clinical decision‐making may benefit from a pragmatic AND‐based combined rule (inclination + Hct), although hematocrit alone remains more efficient (Figure 3); (5) reoperation should be viewed as a retrospective marker of case complexity, not as a prospective stratification factor. As this was a single‐center study conducted in a certified Endoprosthetic Center with standardized perioperative workflows, the findings reflect a controlled high‐volume setting and may not be fully generalizable to institutions with different structures or surgical volumes.

### 4.8 Limitations

Several limitations must be acknowledged: (1) The retrospective design does not allow causal inference and may introduce documentation‐related biases. (2) Missing data were managed by complete‐case analysis, which may reduce power or introduce selection bias. (3) Multicollinearity was not formally assessed despite the inclusion of multiple correlated hematologic parameters and consequently, individual odds ratios—particularly for laboratory variables—should not be interpreted causally, and the regression results should be viewed as exploratory; (4) No internal validation (e.g., bootstrapping or cross‐validation) was performed. Consequently, the reported AUC may overestimate true predictive performance due to model complexity, the high number of predictors relative to transfusion events, and potential overfitting. The discriminative performance should therefore be interpreted as exploratory. (5) Reoperation is a postoperative event and cannot be used for prospective risk stratification; its association with transfusion reflects shared perioperative complexity rather than predictive utility; (6) As this was a single‐center EPZ study, external generalizability may be limited but reflects real‐world standardized workflows in a high‐volume certified environment; (7) Statistical significance of variables with low discriminative performance does not imply meaningful clinical discrimination and should be interpreted with caution; (8) The extremely strong association of the 48‐h hematocrit with transfusion suggests quasi‐separation within the logistic regression model, resulting in numerically extreme odds ratios; therefore, effect sizes should not be interpreted literally; (9) The single‐center design limits external validity, and the findings may not be directly generalizable to non‐certified centers or institutions with different perioperative workflows; (10) Cup inclination measurements were derived from routine postoperative radiographs, and no formal inter‐ or intra‐observer reliability analysis was performed, which may have introduced measurement variability; (11) Although institutional transfusion standards were applied throughout the study period, temporal or physician‐related variability in transfusion decisions cannot be completely excluded due to the retrospective design; (12) All associations identified in the multivariable analysis should be interpreted cautiously, as the model was explicitly exploratory, non‐validated, and not designed for causal inference. Statistically significant predictors are therefore discussed as signals of association rather than definitive determinants of transfusion risk.

## 5 Conclusion

Postoperative 48‐h hematocrit is a strong and clinically actionable marker associated with perioperative transfusion in primary THA. Cup inclination should be interpreted as an indirect proxy of surgical quality rather than an isolated predictor, while reoperation reflects underlying complexity rather than a preoperative risk factor. Hematocrit‐based thresholds may support early postoperative risk stratification for transfusion, whereas inclination‐based cutoffs lack discriminative power. These findings support the use of early postoperative laboratory markers for transfusion risk assessment, rather than isolated radiographic or intraoperative parameters.

## Author Contributions

**Nikolai Ramadanov:** conceptualization, investigation, funding acquisition, writing – original draft, writing – review and editing, visualization, validation, methodology, software, formal analysis, supervision, data curation, project administration, resources. **Dakota Fuchs:** investigation, data curation, methodology. **Maximilian Heinz:** conceptualization, writing – original draft. **Robert Prill:** writing – review and editing, supervision. **Roland Becker:** writing – review and editing, supervision.

## Funding

The authors have nothing to report.

## Conflicts of Interest

The authors declare no conflicts of interest.

## Data Availability

The data that support the findings of this study are available on request from the corresponding author. The data are not publicly available due to privacy or ethical restrictions.
